# The Use of an Errorless Learning Application to Support Re-Learning of (Instrumental) Activities for People Living with Korsakoff Syndrome

**DOI:** 10.3390/jcm11236947

**Published:** 2022-11-25

**Authors:** Roeline Biemond, Erik Oudman, Albert Postma

**Affiliations:** 1 Experimental Psychology, Helmholtz Institute, Utrecht University, 3584 CS Utrecht, The Netherlands; 2 Amsta, Cluster Korsakoff, 1052 LS Amsterdam, The Netherlands; 3 Slingedael Korsakoff Center, Lelie Care Group, 3086 EZ Rotterdam, The Netherlands

**Keywords:** Korsakoff’s syndrome, amnesia, errorless learning, neuropsychological rehabilitation, cognitive rehabilitation

## Abstract

Korsakoff syndrome (KS) is a severe neuropsychiatric syndrome derived from acute thiamine deficiency and concomitant alcohol use disorders. KS patients need lifelong assistance because of the severity of their cognitive problems. In clinical practice and research, errorless learning has proven to be an effective cognitive rehabilitation method for patients with KS. Our study focused on optimizing errorless learning by introducing new software technology to support the training process of errorless learning. Although the benefits of errorless learning for patients with Korsakoff’s syndrome have been thoroughly investigated, it is currently unclear whether new technology could contribute to better learning and maintenance of everyday tasks. Therefore, an errorless learning application was built. This device is a web application and can be used on a tablet, laptop, or smartphone. The application allows clinicians and researchers to insert pictures, videoclips, timers, and audio fragments in the different steps of an errorless learning training plan. This way, the different steps are visible and easy to follow for patients. Moreover, it ensures as a learning method that the training is executed exactly the same way for each and every training. The aim of this study was twofold: to examine whether the use of the errorless learning application is effective, and whether it leads to better results than a regular errorless learning of everyday activities. In total, 13 patients with KS were trained in instrumental activities of daily living by means of the application, and 10 patients were trained with traditional instructions. Results showed an equal improvement for both training methods. Importantly, the technology group could better remember the training when probed at a later moment than the traditional errorless learning group. These results are promising for further development of novel technology to support errorless learning applications in clinical practice.

## 1. Introduction

Korsakoff syndrome (KS) is a chronic neuropsychiatric disorder caused by thiamine deficiency, which is commonly associated with prolonged use of alcohol. KS is characterized by executive dysfunctions and severe learning and memory impairments, which have disastrous effects on daily life functioning [1,2]. Severe memory impairment as a core characteristic for KS primarily consist of declarative memory amnesia [2]. Both anterograde amnesia and a temporally graded retrograde amnesia for both non-personal and autobiographical information is frequently present in patients with KS [3,4].

In contrast to severe declarative amnesia, patients with KS often display spared procedural memory learning [5]. Procedural memory is the nondeclarative memory system underlying percepto-motor and other cognitive skills. Residual procedural learning and memory is often but not always found based on the type of material acquired [6]. In recent reviews, it has been suggested that patients with KS show a maximum procedural learning potential when the task is minimally dependent on other cognitive domains than procedural learning, when feedback is given during the task, and when the task itself is restricted in response options [2,6].

Memory rehabilitation techniques for KS patients have focused primarily on skill learning. One such technique is errorless learning (EL). EL refers to a learning condition that involves the elimination of errors during the learning process [7]. Elimination of errors may be achieved through a variety of means. In the context of memory rehabilitation, EL includes breaking down the targeted task into small, discrete steps or units; providing sufficient models before the client is asked to perform the target task; encouraging the client to avoid guessing; immediately correcting errors; and carefully fading prompts [7,8]. This contrasts with trial-and-error learning in which guessing is encouraged during acquisition [9,10,11]. In a now classic study, Wilson successfully applied EL in the rehabilitation of everyday skills in a small group of elderly subjects, amnesic subjects, and healthy controls. EL was beneficial with a marked positive effect in the amnesic participants [12].

In the last decade, EL applications in KS patients have gained interest. In a first study on EL in KS, Komatsu et al. (2000) showed a superior outcome in learning face-name associations with patients with Korsakoff’s syndrome using errorless learning methods in comparison with the use of trial and error [13]. More recently, several studies have focused on applying EL in the rehabilitation of everyday instrumental skills. Oudman et al. (2013) demonstrated that (re)learning and maintenance of an instrumental activity using errorless learning is beneficial. While initial learning performance in the errorless learning condition was superior, both intervention techniques resulted in similar improvement over eight learning sessions. Importantly, results showed that after one month without training, KS patients in the EL condition retained their ability to perform the task independently, while patients in the TE condition lost this ability [14].

Rensen et al. (2017) observed significant improvements in task performance after EL training in a large sample of KS patients for activities of daily living, chores, mobility, and housekeeping, as judged by the nurses based on observations [15]. In the same study, quality of life improved in the group that received training. Quality of life was significantly increased on eight of the nine subscales in the KS group who participated in an EL training, but not for patients who did not receive training [9,15]. Rensen et al. (2019) found that the same errorless learning training effectively reduced psychotic symptoms and provoked confabulations, affective symptoms, and agitation/aggression in KS patients. There were no significant changes in the control group. Levels of apathy were stable over time in both groups [16].

Given the promising potential of EL in KS patients, a further gain could be made by optimizing the training procedure. One way to do so could be the application of assistive technology. In dementia care, there is a growing interest in electronic assistive technology to support patients [17,18]. The low costs and wide availability of assistive technology makes it attractive to use for treating disabled persons [19]. In the recent literature, the use of electronic devices helping patients diagnosed with KS also received some attention. For example, in a study by Svanberg and Evans (2014), the impact of the SenseCam was investigated in a KS patient. The SenseCam is a wearable, automatic camera, on subjective mood and identity in a patient with severe memory impairment due to Korsakoff’s syndrome. The authors concluded that the SenseCam may be of significant use as a compensatory memory aid for people with KS [20]. Moreover, Lloyd et al. (2019) examined the benefit of a smartwatch and smartphone in prospective memory in patients with KS. Time accuracy was improved with a smartwatch and smartphone condition compared to a situation without assistive technology. Furthermore, the smartwatch and phone conditions were more effective than no aid in assisting memory for task content [21]. Smits and colleagues (2021) examined the possible benefits of a smartwatch aid for prospective memory tasks in patients with KS and compared its efficacy with verbal in-person reminders. The findings suggested that a smartwatch is as beneficial as verbal reminders as an external memory aid for PM tasks in KS patients [22]. Although the benefits of EL for KS patients have been investigated thoroughly, it is currently unknown whether technology could contribute to the training process. To investigate this topic, we created an errorless learning application (ELA) to (re)learn everyday skills based on visual and verbal commands that could guide the patients during the learning process. One of the most profound benefits in using the ELA is standardization, i.e., the assurance that every training session is done strictly the same way. Moreover, the possibilities of training skills and activities could potentially be endless in using this application. Further, it is an innovative and easy method for caretakers to use, as well as for clients to learn or relearn new skills.

The aim of the current study was to examine the effectiveness of errorless learning using an app in a group of Korsakoff patients. In comparison with this experimental group, a patient–control group was trained with verbal EL.

## 2. Methods

### 2.1. Participants

In total, 23 patients diagnosed with Korsakoff syndrome participated in this study. More specifically, 10 patients participated in the regular errorless learning (EL) condition, and 13 patients participated in the ELA condition. All patients were inpatients of ‘Amsta’, Amsterdam, The Netherlands. They resided on 3 specialized long-term care facilities for patients diagnosed with Korsakoff syndrome. To be included in this study, patients had to meet the DSM-5 criteria of alcohol-induced major neurocognitive disorder, amnesic-confabulatory type (291.1) [23], and meet the Korsakoff syndrome criteria outlined by Kopelman [24]. Diagnosis was confirmed by a specialist geriatric medicine and healthcare psychologist. All patients had an average premorbid level of intellectual functioning (>90 WAIS-4 IQ score) [25]. Patients with signs of dementia or a diagnosis of acute psychiatric conditions were not included. The data included in this study were obtained according to the principles of the Helsinki Declaration. Prior to the start of this study patients and their legal representatives gave their written informed consent.

### 2.2. Task and Procedure

In total, 6 caregivers (nurses and activity therapists) from the participating wards were trained in errorless learning principles by the Korsakoff Knowledge Center (Korsakov Kennis Centrum), a Dutch nationwide network organization specialized in the care for patients diagnosed with Korsakoff syndrome. Principles of the errorless learning training were based on publications [9,14] that detailed successful application of errorless learning principles in an everyday Korsakoff patient setting. After this formal training, caregivers were trained to operate an ELA by the first author of this manuscript. In a training session, the caregivers were instructed how to use the application and how to train participants. Each participating patient was randomly given a number, which correlated to either the experimental group (ELA) or the control group (EL).

After random assignment, caregivers trained the participants in instrumental activities of daily living, such as ironing clothes, making coffee, or setting the tables. The individually chosen tasks were mutual agreed upon by the patient and caretaker, based on the wishes of the patient. As the skill was trained by learning a routine, in a specific situation, after onset of cognitive disorders, it was a newly trained routine for all patients.

**Figure 1:**
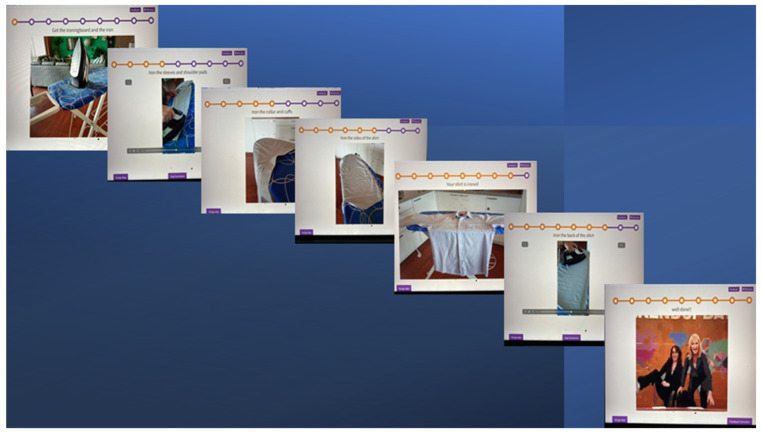
An example of the learning scheme ‘ironing’ as shown in the errorless learning application.

Before the training started, the selected tasks were broken down by the caretaker and psychologist/researcher into small steps, with a recommended maximum of 12 steps per task. For the experimental group, pictures or video clips were made for every step and then loaded into the application (Figure 1 and Table 1). For the control group, the steps were written down.

Before the first session with the patient, the caretaker went through the training of the session with the help of the psychologist/researcher. Every task was trained at least once a week. After each training session, the caretaker was asked to fill in a feedback form. This form enabled the caretaker to indicate which step was done independently, with help or not at all. Patients were trained with a maximum of 10 training sessions, or when a patient was already able to perform the skill after three completely independent training sessions, the training stopped.

Post measurement was done after three months concerning overall contentment with the materials used and the trained task. First, patients were asked if they recognized the materials used (ELA or EL). Patients were also asked about the usability of the materials (ELA and EL). The final question concerned whether they still executed the trained task in daily life.

## 3. Use of the Errorless Learning App

The caretaker opened the application on the iPad and navigated to the specific learning plan the patient was about to train. The patients then received the iPad when the learning plan was opened, as described in the first step (see Table 2).

The caretaker stayed in the room to observe the performance of each step. When a patient needed help or was unable to follow the routine of action, the caretaker prevented making mistakes in the learning process and assisted the patient on that specific step. After, the patient regained control.

Instruction: the caretaker gives the iPad and explains that he/she is able to operate the app him/herself by clicking next step, on the right below.

## 4. Materials

### 4.1. The Desktop Application (ELA)

The ELA was developed to create new possibilities in applying errorless learning principles in patients with Korsakoff’s syndrome and, in doing so, making it easily accessible for patients and caretakers. The main principle of the ELA is that the instructions for the to-be-learned instrumental activity of daily living are given by an application rather than an instructor (Figure 1).

The application runs on a secured internet environment. When opening the application, a therapist can fill in the patient’s name and are then referred to the patient’s ‘page’, with his or her tasks to be learned at that moment, divided into, if needed, morning, afternoon, and night. When the therapist clicks on a task to be learned, it opens the learning scheme and starts with the first step. Right below this page, there is a button titled ‘next step’. This way, one can get to the next step in the training. Each step shows a picture or video clip or in some cases a timer. To underline the picture of video clip, there is a written instruction above the image.

On the top of the page, there is a string with dots. Every dot stands for a step in the learning scheme. The steps already taken are colored dots. This way the patient is able to see which step he or she takes and the total amount of steps, depending on the complexity of the task.

The patient is able to click on the button ‘next step; by him of herself. If a patient still needs help doing that, the caretaker will step in and click on the button. After the last step in the scheme, there is the final step that shows a short celebration video (.gif-file), congratulating the patient with another training session. This final step is used as a motivational cue and positive ending of a session. After the final step, the patient is done training. Instead of the button ‘next step’ appearing, a different button appears. This button says ‘feedback form’. This form is filled in by the caretaker who is present during training. The data of the filled-in form is saved by the application. The back-end of the application is used to create personalized learning schemes. To make a new learning scheme, the button ‘create wizard’ is used. On this page, one determines the different steps of the scheme, with a maximum of 12 steps. The action sequences are transformed into written instruction and an image. For every step, there is the possibility to load a picture or video clip. The application enables the caretaker to personalize the learning scheme. As an example, when a patient is trained to learn to keep a room clean and tidy, pictures and video clips are made of his/her own room. Apart from loading pictures and video clips, a timer can be used. The timer can be useful when something needs a specific time, like brushing teeth. Every step is also underlined by a written instruction. After saving the scheme, it appears on the front side of the app. After the training sessions, the feedback form pops up and data is saved by the application.

### 4.2. The Feedback Form

This form is used in both groups. It is a standard part of the ELA. The control group uses a paper version. The task was broken into small action sequences (see Table 1). The action sequences were transformed into written instruction. This process was supervised by the psychologist/researcher and validated in the team of occupational therapists and nurses who executed the training sessions in this research.

### 4.3. The Post Measurement

Three months after training, patients were asked to fill in a questionnaire concerning contentment of the training (score between 0 (not usable) and 10 (very usable), recognition of the material used (yes or no), and performance of the learned skill in daily life (yes or no)).

### 4.4. The Task Learnt

The trained tasks varied between patients, depending on the wishes of the patient. The tasks varied from setting the tables (2), bathing (1), brushing teeth (2), tidying room (2), tidying up a wardrobe (2) making up a bed (2), ironing (2), doing the laundry (2), making porridge (1), getting coffee (1), cleaning a walker (1), tidy clothes in the closet (2) to sending messages on smartphone, using WhatsApp (2), and sending emails (1).

### 4.5. Data Analysis

The statistical analysis was performed using SPSS Statistics 26. A general linear model repeated measures was performed with the training scores of both groups. Mauchly’s test of sphericity was performed when a violation of sphericity was found. All tests were corrected for multiple comparison.

## 5. Results

### 5.1. Demographic Characteristics

In total, 3 women and 21 men diagnosed with Korsakoff’s syndrome completed the training protocol (Table 3). One patient in the ELA group stopped the training procedure because of motivational issues.

The mean age of the participants was 62 years old, with a range from 46 to 76 years. The ELA group consisted of 13 patients (2 women and 11 men, with a mean age of 66.84 years, in a range of 55 to 76 years). The EL group consisted of 10 patients (1 woman and 9 men, a mean age of 60.2 years, with a range from 46 to 76 years).

The total mean between the group patients in the ELA group and EL group was significant: (*t* (21) = 2.094; *p* = 0.49), suggesting an older age of the ELA group.

### 5.2. Errorless Learning and Errorless Application Learning

**Figure 2:**
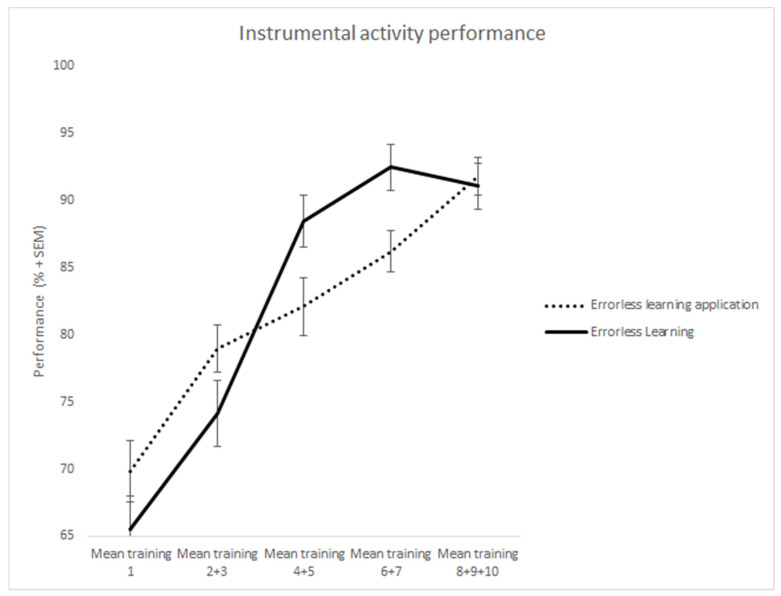
The mean scores within the ELA group and the EL group.

The total group of 23 patients completed 5 training sessions. After the fifth session, some patients fully and successfully completed their training and, thus, the sessions were stopped. To compensate and to make the groups complete, the last score of the trainings sessions was filled for the final sessions. To level out the capriciousness in different measurements within the groups, the measured moments were taken together in a few groups: measurement 1, 2 + 3, 4 + 5, 6 + 7, 8 + 9 + 10 (see Table 4, Figure 2).

Both groups performed equally well on the first learning session: ELA; M = 1.34; SD = 0.37, P&P; M = 1.37; SD = 0.23. (t(14) = 1.1, *p* = 0.291). There were no significant differences found.

To investigate whether errorless learning with the ELA could effectively support the learning of different tasks, performance in 10 learning sessions was examined. This effect was also examined for the traditional errorless learning group. Mauchly’s test of sphericity indicated that the assumption of sphericity was violated for session in the ELA condition, as well as the P&P condition. Therefore, we used Greenhouse–Geisser-corrected values for the results of the repeated measures ANOVA of this variable in both conditions.

A main effect was found for session (F(1.678,3.523) = 18.126, MSE = 1.903, *p* < 0.001, η^2^ = 0.475), indicating that during the learning phase skill performance improved in both groups. There was no significant difference between the groups: F(1,21) = 0.23, *p* = 0.42.

Importantly, the session x group interaction was not significant — F(2.88, 60.6) = 0.13, *p* = 0.44 — indicating that both groups had equal benefits of their learning conditions.

### 5.3. Follow-Up

Two clients in the EL control condition did not participate in the post-measurement because they passed away.

After three months, patients were asked whether they recognized the material used, either ELA (opening screen) or a paper errorless learning plan. Fisher’s exact test was conducted, which showed a significant difference: X2(1) = 0.005; *p* = 0.002. Patients in the ELA group recognized significantly more often the material used in training as opposed to the patients in the EL condition (ELA 9, EL 0).

Patients were also asked after the usability of the material used. They were able to give a score between 1 (not usable at all) and 10 (very useful). An independent sample *T*-test was done and showed a difference in outcome: ELA (M = 6.0, SD = 1.12), P&P (M = 4.6; SD = 1.30). The scores in the ELA group were elevated but there was no significant difference found (*t*(19) = 1.643, *p* = 0.12).

There was no significant difference between the groups in the degree to which patients were still actively using the learned task in everyday life. Fisher’s exact test showed no significant results between the two conditions (*X*^2^(1) = 1.00, *p* = 0.502). Out of the 13 patients, 9 in the ELA group continued to carry out the trained skills, whereas 7 of 8 in the traditional EL group continued to carry out the trained skills.

## 6. Discussion

The aim of this study was to examine the effectiveness of the use of an errorless learning application (ELA) in comparison to regular errorless learning (EL) in the rehabilitation of everyday skills in KS. Our results show a clear positive effect of both techniques in (re)learning an everyday skill. Within 5 training sessions, most of the patient mastered a new skill up to a rather adequate level. Both learning methods were equally effective. Of importance, the errorless learning application led to better recognition of the learned material.

We replicated the finding that patients diagnosed with KS can successfully learn an everyday task by means of errorless learning principles [9,14,15]. Both the traditional training technique applied in EL, and the use of the ELA resulted in relatively fast mastery of a formerly unavailable skill. Our findings also demonstrate the usefulness of an errorless learning application in clinical practice, hereby possibly reducing the time and resources for successful implementation of errorless learning.

A prior study of Oudman et al. (2013) showed a notable outcome of the long-term effect of errorless learning. Patients trained with errorless learning still executed their trained skill after one month as opposed to the trial-and-error trained group [14]. In our study, patients in the errorless learning application group still performed the task as frequently as in the traditional errorless learning group, but patients could still remember using the application. Both results show the finding of applying errorless learning successfully in a clinical context. We may mention here that app-related cues in this recognition were more elaborate and visual than those used for the paper-and-pencil condition. This also partly could explain the better recognition performance. Cascella en Khalili (2021) elucidated three pathways in memory encoding: visual, acoustic, and semantic encoding. These processes interact with each other, and diverse brain structures register the original episode in an organized way memory traces [26]. These observations illustrate that training by means of the app might generate a stronger or better accessible memory trace for the learning episodes and task contents.

Earlier studies showed positive results in the use of technical aids in memory rehabilitation for patients with KS [20,21,27]. This is the first study to examine the effect of using an ELA to facilitate the learning process. It corroborates the outcome of studies in the effectiveness in using a technical aid in memory rehabilitation. Importantly, results concerning contentment with the training showed a difference in mean score. Although no significant difference was found, patients trained in the ELA group showed an elevated mean score. This is possibly a promising result, because it shows that patients are not only capable of using new technology in cognitive rehabilitation but that they may also value it more positively. The height of the mean score may be affected by the effect size of this study.

Contemplating the results there were a few other factors which may have limited the outcomes of the application-based training. The application used in this research was a first version of the application. The basic application mainly focused on the different errorless learning schemes, aiming to address the patients and not necessarily the caretaker who trained the patients. During this study, it became clear that the current application lacked some functions in ease of use for caretakers, such as a visible insight of the learning specifics of the patient trained and the possibility of exchanging feedback and suggestions among the caretakers who were training the patients. The most notable observation was the reluctance of some caretakers in training with the application. A recent software update resolved many of the earlier issues. These new functions may make the application more accessible for caretakers using the application in training with patients.

Because the training collided sometimes with the standard clinical care given to patients, we were unable to collect a richer data set. For pragmatic reasons, other possible measures such as quality of life of the patient, job satisfaction of the caregiver, and motivation of the patients were not included in order to reduce the workload for the caregivers participating in this research. Although there were no extra measurements included, the results are of great clinical importance to continue the research. Introducing technology on different wards is something new, which caretakers and other disciplines have to get used to. It takes time to get familiar with. In retrospect, this process was underestimated when starting this research. Even though at first the use of the ELA was intended to be used for research purposes, it should have been more widely supported throughout different disciplines at the wards. It would have been easier for caretakers to get used to this new way of using errorless learning while being supported by different CEOs executives and disciplines on the wards.

This study has a number of limitations. The amount of patients participating in the training was unfortunately limited. Many studies in KS research are relatively underpowered. Because the population of KS patients worldwide is relatively small, this limitation seems somewhat inherent to KS studies. Results show a significant age difference between the two groups. Caretakers and therefore patients were randomly assigned to a group. Due to drop out, age differences occurred. Because the age differences are relatively minimal, and scores were comparable on the first learning session, we determined that the influence of this age difference was limited. The setup of this study consisted of two groups trained with errorless learning. There was no control group using for example trial and error learning. Although multiple studies point to a beneficial effect of EL in KS, good case-controlled studies are still missing. It would be relevant in future research to investigate EL in a large group of KS patients with a pre-defined control condition. Another limitation is the influence of a treatment effect. The caretakers participating in this study were all trained the same way and had the same positive expectations in training with errorless learning. As shown in a study of Rensen et al. (2017) [9] concerning quality of life after EL, which results showed a very positive effect on quality of life, there is a possibility that these positive effects in both studies can be partly explained by an effect of treatment, where positive effects are expected by the caretakers. A strength of the present study is that it is the first study to our knowledge to apply assistive technology on errorless learning in this population of participants. Moreover, this application forms an easier way to implement errorless learning into clinical practice with possible benefits for multiple populations in clinics and at home. Furthermore, both patients and caretakers were very enthusiastic regarding the usability of the ELA. The use of the application creates new possibilities and advantages in clinical practice as well as research purposes. A general advantage is the certainty that every training session is done the same way as patients all follow the steps shown on the screen. In context of research, it creates endless possibilities of studies in fine tuning errorless learning combined with visual components. In the near future, the application could possibly also be very helpful for patients with KS in less intensive forms of care or at home.

In conclusion, the results of the present study indicate that Korsakoff’s syndrome patients are able to (re)learn and maintain an instrumental activity by using an errorless learning application. Moreover, patients in the ELA group did have better recognition for the training materials and were more content with the materials and training format. Importantly they also tended to practice the trained skills more often in comparison with de patients in the group trained with pencil and paper. Taken together the results clearly show that ELA is an inspiring technique that deserves further research.

## Figures and Tables

**Table 1:** An example of the results on the learning scheme ‘ironing,’ as shown in the errorless learning application.

Training Session	Results
1.	3 steps independent; 5 steps with help
2.	3 steps independent; 5 steps with help
3.	2 steps independent; 5 steps with help; 1 step unable
4	4 steps independent; 3 steps with help; 1 step unable
5	3 steps independent; 4 steps with help
6.	7 steps independent; 1 step with help
7.	7 steps independent; 1 step with help *

* The patient continued to need some help with step 1 to get the board and iron out of the closet.

**Table 2:** Errorless learning plan for ‘ironing’.

Step 1	Cue in application: get the iron board and iron (picture) Cue in application: next step
Step 2	Cue in application: put the power plug of the iron in the electrical outlet (picture) Cue in application: next step
Step 3	Cue in application: get the shirt and place it on the ironing board (picture) Cue in application: next step
Step 4	Cue in application: iron the sleeves and shoulder pads (videoclip) Cue in application: next step
Step 5	Cue in application: iron the collar and cuffs (picture) Cue in application: next step
Step 6	Cue in application: iron the sides of the shirt (picture) Cue in application: next step
Step 7	Cue in application: iron the back of the shirt (videoclip) Cue in application: next step
Step 8	Cue in application: your shirt is ironed Cue in application: next step
Step 9	Cue: celebrating GIF for a positive ending of the training session and to create motivation in training a next time.

**Table 3:** (**a**) Mean age of Korsakoff’s syndrome patients in the errorless learning application (ELA) and errorless learning (EL) groups. (**b**) Mean total age of Korsakoff’s syndrome patients in the errorless learning application (ELA) and errorless learning (EL) groups.

(**a**)			
	**N**	**Mean**	**Standard Deviation**
ELA	M (11)	66.18	8.36
	V (2)	70.50	0.70
EL	M (9)	61.78	5.47
	V (1)	46.00	-
(**b**)			
	**N**	**Mean**	**Standard Deviation**
ELA	13	66.84	7.80
EL	10	60.20	7.17

**Table 4:** The mean scores and standard deviation of the instrumental activity performance within the ELA and EL group.

		Mean	SD	N
Training session 1 mean score	Errorless learning application	1.34	0.32	13
	Errorless learning pencil and paper	1.37	0.22	10
	Total	1.35	0.31	23
Training sessions 2 and 3	Errorless learning application	1.53	0.28	13
	Errorless learning pencil and paper	1.53	0.28	10
	Total	1.53	0.27	23
Training sessions 4 and 5	Errorless learning application	1.63	0.30	13
	Errorless learning pencil and paper	1.79	0.24	10
	Total	1.70	0.28	23
Training sessions 6 and 7	Errorless learning application	1.73	0.21	13
	Errorless learning pencil and paper	1.85	0.22	10
	Total	1.78	0.22	23
Training sessions 8–10	Errorless learning application	1.83	0.19	13
	Errorless learning pencil and paper	1.85	0.22	10
	Total	1.84	0.20	23

SD = Standard Deviation, N = number of participants.

## Data Availability

Data supporting results are saved at the server of the University of Utrecht. Due to patient confidentiality, raw data are not made publicly.

## Abbreviations

The following abbreviations are used in this manuscript:

KS	Korsakoff’s syndrome
EL	errorless learning
ELA	errorless learning application
